# Supplementary material for: Iterative improvement in the automatic modular design of robot swarms
Source: PeerJ Comput Sci. 2020 Dec 7;6:e322. doi: 10.7717/peerj-cs.322 (PMC7924708; doi:10.7717/peerj-cs.322)
Supplement: Supplemental Information 3 [file peerj-cs-06-322-s003.zip › argos3/doc/api/standalone/a00415_source.html]

ARGoS: plugins/robots/e-puck/simulator/epuck\_entity.h Source File


- Main Page
- Related Pages
- Namespaces
- Classes
- Files

- File List
- File Members

# plugins/robots/e-puck/simulator/epuck\_entity.h

Go to the documentation of this file.

```
00001 
00007 #ifndef EPUCK_ENTITY_H
00008 #define EPUCK_ENTITY_H
00009 
00010 namespace argos {
00011    class CControllableEntity;
00012    class CEmbodiedEntity;
00013    class CEPuckEntity;
00014    class CGroundSensorEquippedEntity;
00015    class CLEDEquippedEntity;
00016    class CLightSensorEquippedEntity;
00017    class CProximitySensorEquippedEntity;
00018    class CRABEquippedEntity;
00019 }
00020 
00021 #include <argos3/core/simulator/entity/composable_entity.h>
00022 #include <argos3/plugins/simulator/entities/wheeled_entity.h>
00023 
00024 namespace argos {
00025 
00026    class CEPuckEntity : public CComposableEntity {
00027 
00028    public:
00029 
00030       ENABLE_VTABLE();
00031 
00032    public:
00033 
00034       CEPuckEntity();
00035 
00036       CEPuckEntity(const std::string& str_id,
00037                    const std::string& str_controller_id,
00038                    const CVector3& c_position = CVector3(),
00039                    const CQuaternion& c_orientation = CQuaternion(),
00040                    Real f_rab_range = 0.8f,
00041                    size_t un_rab_data_size = 2);
00042       
00043       virtual void Init(TConfigurationNode& t_tree);
00044       virtual void Reset();
00045       virtual void Destroy();
00046 
00047       virtual void UpdateComponents();
00048       
00049       inline CControllableEntity& GetControllableEntity() {
00050          return *m_pcControllableEntity;
00051       }
00052 
00053       inline CEmbodiedEntity& GetEmbodiedEntity() {
00054          return *m_pcEmbodiedEntity;
00055       }
00056 
00057       inline CGroundSensorEquippedEntity& GetGroundSensorEquippedEntity() {
00058          return *m_pcGroundSensorEquippedEntity;
00059       }
00060 
00061       inline CLEDEquippedEntity& GetLEDEquippedEntity() {
00062          return *m_pcLEDEquippedEntity;
00063       }
00064 
00065       inline CLightSensorEquippedEntity& GetLightSensorEquippedEntity() {
00066          return *m_pcLightSensorEquippedEntity;
00067       }
00068 
00069       inline CProximitySensorEquippedEntity& GetProximitySensorEquippedEntity() {
00070          return *m_pcProximitySensorEquippedEntity;
00071       }
00072 
00073       inline CRABEquippedEntity& GetRABEquippedEntity() {
00074          return *m_pcRABEquippedEntity;
00075       }
00076 
00077       inline CWheeledEntity& GetWheeledEntity() {
00078          return *m_pcWheeledEntity;
00079       }
00080 
00081       virtual std::string GetTypeDescription() const {
00082          return "e-puck";
00083       }
00084 
00085    private:
00086 
00087       void SetLEDPosition();
00088 
00089    private:
00090 
00091       CControllableEntity*                   m_pcControllableEntity;
00092       CEmbodiedEntity*                       m_pcEmbodiedEntity;
00093       CGroundSensorEquippedEntity*           m_pcGroundSensorEquippedEntity;
00094       CLEDEquippedEntity*                    m_pcLEDEquippedEntity;
00095       CLightSensorEquippedEntity*            m_pcLightSensorEquippedEntity;
00096       CProximitySensorEquippedEntity*        m_pcProximitySensorEquippedEntity;
00097       CRABEquippedEntity*                    m_pcRABEquippedEntity;
00098       CWheeledEntity*                        m_pcWheeledEntity;
00099    };
00100 
00101 }
00102 
00103 #endif
```

---

Generated on 10 Jul 2018 for ARGoS by 
 1.6.1
